# Multidisciplinary Treatment for Childhood Obesity: A Two-Year Experience in the Province of Naples, Italy

**DOI:** 10.3390/children9060834

**Published:** 2022-06-04

**Authors:** Francesca Gallè, Giuliana Valerio, Ornella Daniele, Valentina Di Mauro, Simone Forte, Espedita Muscariello, Roberta Ricchiuti, Serena Sensi, Mario Balia, Giorgio Liguori, Pierluigi Pecoraro

**Affiliations:** 1 Department of Movement Sciences and Wellbeing, University of Naples “Parthenope”, Via Medina 40, 80133 Naples, Italy; giuliana.valerio@uniparthenope.it (G.V.); giorgio.liguori@uniparthenope.it (G.L.); 2 Nutrition Unit, Department of Prevention, Local Health Authority Napoli 3 Sud, Torre del Greco, Via Montedoro 47, 80059 Naples, Italy; ornyda@yahoo.it (O.D.); dimaurov@hotmail.com (V.D.M.); simoneforte1980@gmail.com (S.F.); edy.muscariello@gmail.com (E.M.); robertaricchiuti@hotmail.com (R.R.); serena.sensi@libero.it (S.S.); mariobalia94@gmail.com (M.B.); p.pecoraro@aslnapoli3sud.it (P.P.)

**Keywords:** childhood obesity, body mass index, body composition, ambulatory care

## Abstract

Childhood obesity must be faced through an integrated multi-level preventive approach. This study was aimed at assessing the adherence and the outcomes of an outpatient service for childhood obesity treatment activated in the province of Naples, Italy, throughout a 2-year follow-up period. At first visit (T_0_), weight, height, waist circumference, and body composition of children were assessed, together with sociodemographic features and physical activity levels of children and parents. Anthropometric and body composition parameters of children were measured at 6 ± 3 months (T_1_) and 12 ± 3 months (T_2_). A total of 451 non-related children who accessed the service were analyzed: 220 (48.7%) of them returned at least once (attrition rate 51.3%). Returner outpatients showed higher age (*p* = 0.046) and father’s educational level (*p* = 0.041) than non-returner ones. Adherence to the treatment was found to be related to father’s (Rho = 0.140, *p* = 0.005) and mother’s (Rho = 0.109, *p* = 0.026) educational level. All the outcomes improved between T_0_ and T_1_ (*p* < 0.001), while only body mass index (BMI) decreased significantly at T_2_. Changes in BMI-SDS were associated with baseline value (OR 0.158, 95%CI 0.017–0.298, *p* = 0.029). The multidisciplinary approach seems to be promising to treat childhood obesity in this geographic context. Lower parents’ educational level should be considered as an attrition determinant.

## 1. Introduction

Currently, childhood obesity represents a public health challenge, due to its negative effects on the health of children and adolescents affected and its role in determining obesity and related morbidity and mortality in adulthood [1]. Several demographic, genetic, and behavioral factors, such as socioeconomic factors, have been identified as determinants of pediatric obesity. In particular, pediatric obesity is strongly associated with an inadequate balance between energy intake and expenditure in children and adolescents, which in turn strictly correlates with parental weight status, attitudes and behaviors towards food and physical activity (PA), and with an obesogenic environment which favors these unhealthy lifestyles [2,3]. Childhood obesity can have immediate negative effects on children’s physical and academic performances and cause a higher risk for several comorbidities in the later life, such as dyslipidemia, hypertension, non-alcoholic fatty liver disease, coronary heart disease, and type 2 diabetes [4,5].

In the last four decades, the number of obese children and adolescents has risen tenfold worldwide, with negative future perspectives on global health [6]. In Europe, the analysis of the WHO European Childhood Obesity Surveillance Initiative (COSI) data collected between 2007 and 2013 show that one in four children with obesity were severely obese and that the prevalence of severe obesity varied greatly among the 21 countries involved, with higher values in Southern Europe [7]. In Italy, the surveillance system “OKkio alla Salute” from the Ministry of Health reports a nationwide prevalence of 9.4%, with the Campania region accounting for the highest regional value (12.6%) [8]. This situation may have been worsened by the increased adoption of unhealthy behaviors during the current COVID-19 pandemic [9].

Given its complex etiology, an integrated multi-level approach is necessary to treat childhood obesity. According to the World Health Organization recommendations, primary healthcare services are important for the early detection and management of obesity and its associated complications in children and adolescents. Subsequently, appropriate family-based, multicomponent (including nutrition, physical activity, and psychosocial support) weight management services, delivered by multiprofessional teams with appropriate training and resources, should be offered as part of universal health coverage following a staged approach [10,11]. Specifically, in Italy the second-level center defines the clinical condition of children referred by the primary care pediatricians and runs the multidisciplinary intervention that is centered on diet education and lifestyle modification. Third-level centers admit patients who are suspected of secondary obesity, showed no response to the second-level treatment, or severe comorbidities, compromised psychological balance, or significantly impaired quality of life that requires more specialistic diagnostic assessment and/or intensive care programs, including bariatric surgery [12].

In March 2018, a second-level outpatient service for childhood obesity treatment was activated in the province of Naples, county town of the Campania region, south Italy. It is based on a family-based approach by which outpatients receive psychological support and nutritional and physical activity counseling.

This study was aimed to analyze retrospectively the adherence to treatment and the anthropometric and body composition outcomes produced in the adherent outpatients throughout a 2-year observation period.

## 2. Materials and Methods

### 2.1. Participants and Setting

Data from children and adolescents seeking obesity treatment at the outpatient clinic “Second Level Assistance Center for Diabetes and Obesity in Childhood” of the Local Health Authority “Napoli 3 Sud” were retrospectively analyzed in this study. Children aged 5–14 years with diagnosed obesity are addressed from their primary care pediatrician to the Center. For the purposes of this study, those who received their first visit (indicated as T_0_) between 2018 and 2020 were consecutively included, and two follow-up times were considered: 6 ± 3 months (T_1_) and 12 ± 3 months (T_2_). Children who returned to at least one follow-up visit were defined as “returners”. At admission, parents or guardians of the outpatients signed an informed consent to the use of their personal information.

Sociodemographic features of children and parents were collected at first visit through parent/guardian interviews. Parents’ educational level was assumed as a proxy indicator for socioeconomic status.

### 2.2. Intervention and Outcomes

The multidisciplinary intervention consists of counseling sessions about nutrition, PA, and motivational support carried out by a multidisciplinary team (nutritionists, kinesiologists, and psychologists). The counseling is performed in a single day and in the presence of parents; it lasts about 1 h and 45 min and is structured in three phases. In the first thirty minutes, the nutritionist discusses with the outpatient and their parents/guardians to collect personal and anamnestic data as well as eating habits of the outpatient and their family. In particular, dietary assessment, aimed to investigate energy intake and eating habits, is performed by using a food frequency questionnaire [13]. Successively, the specialist measures the anthropometric parameters and assesses the body composition through bioimpedance analysis. Height is measured to the nearest 0.1 cm by using a stadiometer (Wunder, Milan, Italy) [14]. Body weight, recorded in kilograms and rounded to the nearest 1 decimal point, is measured using a platform scale (Wunder, Milan, Italy) [15]. Weight and height are assessed by the same investigator, specifically trained. The body mass index (BMI) is calculated and expressed in kg/m^2^, and then it is converted into BMI standard deviation score (BMI-SDS) [16]. BMI/age growth percentiles are used to define the patient’s degree of obesity according to the World Health Organization cut-offs [17,18]. Waist circumference (WC) is measured through a measuring tape (Seca, Hamburg, Germany) as a proxy of visceral fat and expressed in centimeters. Body composition is assessed by bioelectrical impedance analysis (DS Medica, Milan, Italy). The analysis is carried out according to hand to foot method. In particular, tetrapolar hand-foot measurement is performed on the supine subject for 15 min, placing gel-filled electrodes at the metacarpal and metatarsal joints of the phalanges [19]. Fat mass (FM) and fat free mass (FFM) are expressed either in percentages or kilograms.

Thereafter, the psychologist supports outpatients and their families in changing nutrition and lifestyle habits detecting their own physical, psychological, and relational resources. The psychological visit (45 min) is aimed to structure an adequate motivation in changing lifestyle. It consists of a psychological welcome interview with the whole family unit aimed to detect the relational dynamics and the context in which the patient grows up, to analyze the intrapsychic area (psycho-affective development, relationship with food, time of obesity onset, stressful/traumatic events), to investigate the social area (socializing activities, use of free time, interpersonal relationships), to evaluate the motivation to change, and to perceive any psychological aspects related to eating behavior (loss of control over food, body image alterations, difficulty in processing and containing negative emotions, etc.). Motivational counseling sessions are scheduled in case of poor compliance with the treatment and/or severe obesity and/or relationship difficulties [20]. In particular, a group/individual/family counseling session is carried out, according to the patient’s age and the type of emerged problems, to dissolve the individual and/or family troubles that reinforce obesity, hindering the internalization of healthy lifestyles. Motivational counseling consists of a cycle with 4 weekly meetings which ends with a return to the parents of the emerged contents [21].

In the last 30 min, the kinesiologist estimates physical and sedentary activities of both patients and their parents. The Physical Activity Questionnaire for Children (PAQ-C) [22] is administered to the former, while the International Physical Activity Questionnaire-Short Form (IPAQ-SF) is administered to the latter [23]. In addition, during the first visit, the kinesiologist sets out the benefits of healthy lifestyle and the strategies to increase the daily physical activity levels for the whole family [24] and to reduce screen time and sedentary behaviors, in a context of empathy and support [25]. Furthermore, an adapted physical activity protocol is prescribed based on specific parameters such as frequency, intensity, time, and type [26].

Lastly, a personalized balanced nutritional scheme, according to the individual nutritional history, caloric requirement, resting energy expenditure, and physical activity, is developed for each subject and released to outpatients and their families [27]. A multidisciplinary visit with outpatients and their parents is carried out every month to strengthen lifestyle changes, motivate patients to continue their programs, and highlight possible difficulties related to the path.

The study was approved by the Local Health Authority “Napoli 3 Sud” of Naples (Deliberation n. 92 of 31.01.2020).

### 2.3. Statistical Analyses

A descriptive analysis was performed on sociodemographic, anthropometric, and body composition characteristics and PA levels of the sample and on PA levels of their parents. Categorical variables were reported as number and percentage of subjects, while all the other variables were expressed as median values and interquartile ranges, having assessed their non-normal distribution. The chi-squared test was used to assess differences in gender and educational level of parents between returners and non-returners. The Mann–Whitney U test was employed to compare median values of age and anthropometric and body composition characteristics. A Spearman correlation analysis was performed to highlight possible relationships between sociodemographic and behavioral features of parents and baseline children parameters. The Wilcoxon signed rank test was used to evaluate changes that occurred in compliant patients between baseline (T_0_) and first (T_1_) or second (T_2_) follow-up. A Spearman correlation was used to assess possible correlations between baseline characteristics of the sample, attending follow-up visits, and BMI/BMI-SDS changes at follow-ups. The Mann–Whitney test was used to compare the baseline characteristics of returners who showed or not an increase in BMI-SDS at T_1_ or at T_2_. The chi-squared test was used to analyze the differences in parents’ educational level of outpatients who attended the Center for one, two, or three visits. A linear regression was carried out considering changes in BMI-SDS as outcomes and baseline characteristics as independent variables. The length of follow-up was included as a covariate. Results were reported as Odds Ratios and Confidence Interval 95% (OR, 95%CI).

A value of *p* = 0.05 was considered as level of significance. All the statistical analyses were performed through the IBM SPSS software v. 27.

## 3. Results

Of a total of 451 children and adolescents observed who accessed the Center for the first time, 220 (48.7%) (“returners”) returned for at least a follow-up appointment: 141 (64.1%) attended only one control visit and 79 (35.9%) attended two appointments in the observation period.

Table 1 shows the baseline characteristics of children who returned or not to the Center.

Significant differences between the two groups were detected for age and father’s educational level.

In the correlation analysis performed at baseline, father’s educational level was related with BMI (Rho = −0.233, *p* < 0.001), WC (Rho = −0.183, *p* < 0.001), WC/H (Rho = −0.212, *p* < 0.001), FM (%) (Rho = −0.112, *p* = 0.028), FM (kg) (Rho = −0.144, *p* = 0.005), FFM (%) (Rho = 0.126, *p* = 0.013), FFM (kg) (Rho = −0.115, *p* = 0.023), and BMI-SDS (Rho= −0.206, *p* < 0.001); mother’s educational level was related with BMI (Rho = −0.256, *p* < 0.001), WC (Rho = −0.192, *p* < 0.001), WC/H (Rho = −0.263, *p* < 0.001), FM (%) (Rho = −0.165, *p* = 0.001), FM (kg) (Rho = −0.175, *p* < 0.001), FFM (%) (Rho = 0.179, *p* < 0.001), and BMI-SDS (Rho = −0.191, *p* < 0.001). In addition, CPAQ score was found to be related with FM (kg) (Rho = −159, *p* = 0.035) and parents’ IPAQ score (Rho = 0.189, *p* = 0.011).

Table 2 reports the changes in anthropometric parameters and body composition that occurred in adherent children throughout the period of observation.

The comparisons between anthropometric parameters and body composition values registered at T_0_ and T_1_ showed significant improvements in all the parameters. Similarly, significant improvements were found for anthropometric (BMI, BMI-SDS, WC, and WC/H) variables but not for the body composition variables between T_0_ and T_2_. Only BMI continued to decrease significantly between T_1_ and T_2_.

**Figure 1:**
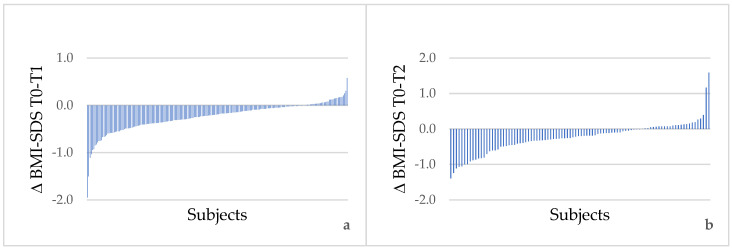
BMI-SDS changes between T0 and T1 for each patient who attended the Center at 6 ± 3 months (**a**) and at 12 ± 3 months; (**b**) after the first visit.

The individual changes in BMI-SDS that occurred in children and adolescents who underwent a second and a third visit are shown in Figure 1a,b, respectively.

An increase in BMI-SDS was registered in 32 out of 205 (15.6%) patients at T_1_ and 24 out of 94 (25.5%) patients at T_2_. In the comparison between baseline characteristics of patients who increased their BMI-SDS and those who did not, only baseline WC values differed significantly (median, IQR 93.5, 91–96 in BMI-SDS maintainers vs. 85.5, 82–91 in increasers; *p* = 0.006).

**Figure 2:**
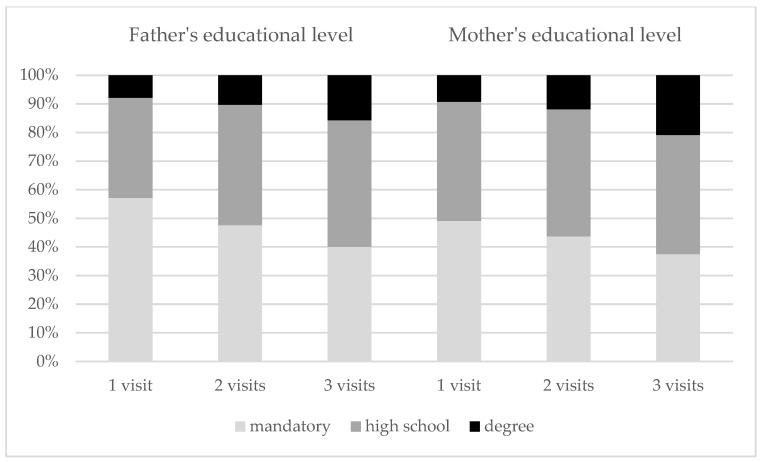
Percent distribution of outpatients’ adherence for father’s and mother’s educational level.

In the correlation analysis, adherence to treatment was found to be related with father’s (Rho = 0.140, *p* = 0.005) and mother’s (Rho = 0.109, *p* = 0.026) educational level. Figure 2 shows the differences in parents’ educational level of outpatients who attended the appointments once (first visit), twice, or three times in the 2-year period (*p* = 0.082 and *p* = 0.096 for father’s and mother’s educational level, respectively).

BMI changes between T_0_ and T_1_ were related with age (Rho = −0.138, *p* = 0.040), initial BMI (Rho = −0.163, *p* = 0.010), WC (Rho = −0.147, *p* = 0.021), FM (kg) (Rho = −0.164, *p* = 0.010), FFM (%) (Rho = 0.137, *p* = 0.032), and parents’ IPAQ score (Rho = 0.230, *p* = 0.041); BMI changes between T_0_ and T_2_ were related with age (Rho = −0.249, *p* = 0.014), initial BMI (Rho = −0.264, *p* = 0.009), WC (Rho = −0.318, *p* = 0.001), FM (kg) (Rho = −0.243, *p* = 0.016), and FFM (kg) (Rho = −0.305, *p* = 0.002). No correlations were found regarding BMI-SDS changes and the baseline variables.

In the regression analysis, only baseline BMI-SDS was associated with changes in BMI-SDS at follow-up (OR 0.158, 95%CI 0.017-0.298, *p* = 0.029).

## 4. Discussion

This study shows the main characteristics and preliminary outcomes of a multidisciplinary approach to childhood obesity treatment implemented in a region of southern Italy with a high prevalence of childhood obesity. The results suggest that this type of intervention may be promising to improve anthropometric parameters among children with obesity in the geographical area studied.

In the last decades, a growing body of literature has analyzed the outcomes of multicomponent interventions aimed at treating childhood obesity [28]. The components, the organization, and the length of these interventions vary across the studies [29]. However, evidence highlights that combined behavioral lifestyle interventions can significantly reduce the prevalence of overweight children and adolescents compared to standard care or self-help [28]. Notwithstanding outcomes of similar experiences may be appreciated even in the short term [30,31], some of them may result durable even in the long term [32].

Moreover, beyond specific experimental experiences, real life requires that easy to manage, accessible, and sustainable services are offered to people with obesity and their families. In light of this, we examined the performance of the first service implemented in a territory with a higher prevalence of childhood obesity. Our follow-up analysis allowed to observe retrospectively the participation in the program throughout two years. A consistent dropout was observed, since more than the half of the admitted outpatients did not continue the treatment offered to them [33]. Different determinants may act at societal, health system, and service levels, influencing the engagement in healthy lifestyle programs [34]. Furthermore, at the individual level, high attrition rates can be caused by previous negative healthcare experiences, low satisfaction towards the intervention, insufficient perception of its effectiveness, or family barriers to participation such as lack of time or logistical difficulties, perceived costs of healthy food, and lack of exercise opportunities [35,36]. In addition, it should be considered that a part of this study covered the first months of the COVID-19 epidemic spread in Italy, which could have affected the participation to the follow-up. In fact, even though during those months a telehealth intervention was performed by the Center to continue the treatment, some outpatients avoided attending the Center to limit their chances of exposure to virus transmission [37]. Although the reasons for dropouts have not been assessed in this study, it should be noted that returners showed significantly higher age and father’s educational level than non-returners. Parents’ educational level was found to be associated with adherence in the subsequent correlation analysis, suggesting that this aspect may have acted as a determinant for the attrition rate, according to the study by Gunnarsdottir et al. [38]. Even though the psychoeducational intervention aimed to highlight the importance of adhering to a healthy lifestyle is modulated according to the sociocultural characteristics of the patient, this finding suggests that the economic, social, and cultural context in which the child and his/her family live should be more considered, providing targeted information, especially in the case of families in modest social conditions.

As for the treatment outcomes, we assessed a reduction in BMI-SDS in the greatest part of children and adolescents who continued to attend the outpatient service. In the correlation analyses, an increase in BMI was negatively associated with age and the extent of BMI at baseline, and BMI-SDS growth was negatively related with baseline value in the regression analysis, contrary to other studies reporting that younger and less overweight children particularly benefit from lifestyle interventions [39,40]. Furthermore, BMI-SDS changes were more likely to be maintained/decreased in patients who had a greater baseline WC. This is in contrast with the findings of previous studies, who reported larger initial waist circumference as a negative predictor of BMI-SDS reduction in lifestyle interventions for obesity treatment, suggesting the need for addressing more intensive interventions with obese children with specific abdominal fat distribution [41,42]. In agreement with systematic research on the predictors of weight loss and body weight loss maintenance in children with obesity, pre-existing physical activity did not have great impact on the success of the therapy [43].

Notwithstanding the discrepancy with other experiences, these associations show that, among our returner outpatients, better outcomes were obtained from those who presented a worse initial condition. This may strengthen the importance of the service offered to the population of the area, which also shows the higher prevalence of severe obese children among the Italian regions [8].

Our findings highlight differences in the participation in the outpatient treatment, such as in the adherence to the treatment components. However, the available data were not sufficient to clearly define the determinants of these differences. The literature suggests that a unique approach for treating obesity in children and adolescents does not exist [40]. Therefore, multidisciplinary programs should be matched to individual needs, focusing on long-term outcomes. This can help to overcome socioeconomic or racial/ethnic disparities. Moreover, a wider approach that goes beyond the clinical setting and involves a broader societal context with the availability of healthy food and nutritional environments, playgrounds, and sports clubs, as well as neighborhood walkability, is also fundamental to make lifestyle changes possible [40].

Beyond these aspects, it should be accepted that lifestyle interventions can be ineffective for some individuals due to biological mechanisms, such as genetics, hormonal changes, adaptive thermogenesis, and neural factors, which undermine weight loss effects and promote weight regain [44]. Therefore, our study highlights the difficulties that may be encountered in weight loss and weight loss maintenance during the implementation of a lifestyle multicomponent treatment for children with obesity. It points out the need to design more effective approaches to prevent weight regain.

This study has several limitations. First of all, the retrospective nature of the study and the lack of a control group limit the validity of its findings with respect to controlled trials. Moreover, some possible determinants such as family income or parents’ behaviors were not explored in depth. The possible association of adherence and intervention outcomes with these factors is therefore lacking. Furthermore, the reasons for non-attending and dropouts were not explored. Analyzing these reasons could have given important indications on the acceptance of the intervention. Further research considering these items is needed. Nevertheless, this study reports the results of a treatment program offered to children and adolescents in a geographical area widely affected by childhood obesity and highlights some important points for its progression and for the implementation of further interventions in this context. Moreover, the objective assessment of anthropometric outcomes and the use of bioelectrical impedance analysis to evaluate body composition in the outpatients strengthens the validity of the study.

## 5. Conclusions

The findings of this study suggest that a multidisciplinary approach to childhood obesity treatment may be feasible to improve anthropometric parameters in children and adolescents with obesity in the local context examined. However, the stability of these outcomes can be obtained through time and with respect to that compliance to the intervention plays a pivotal role. Since a high dropout rate was obtained in the two years observed, it is important to address the outpatients’ attrition by identifying those factors that may hinder their participation. In addition, further research related to the maintenance of weight loss among youths with obesity participating in intervention programs is needed.

In this study, lower parents’ educational levels were associated with lower compliance. In a public health perspective, social support is needed to counteract the possible influence of inequalities and other factors on treatment attrition.

## Figures and Tables

**Table 1:** Baseline sociodemographic, anthropometric, and behavioral characteristics of subjects who returned or not at the follow-up appointments.

Variable	Non-Returners *n* = 253	Returners *n* = 220	*p*
Gender			0.832
male	162 (53.5)	150 (54.3)	
female	141 (46.5)	126 (45.7)	
Age years (mean, range)	10.0 (9.5–10.7)	10.6 (9.9–11.1)	0.046
Father’s educational level			0.041
mandatory	116 (57.1)	88 (44.9)	
high school	71 (35)	84 (42.9)	
degree	16 (7.9)	24 (12.2)	
Mother’s educational level mandatory high school degree	106 (49.1) 90 (41.7) 20 (9.3)	86 (41.5) 90 (43.5) 31 (15)	0.118
Weight (kg) median (IQR)	58.1 (7.9)	62.9 (9.2)	0.326
Height (m) median (IQR)	1.5 (0.1)	1.5 (0.1)	0.237
BMI (kg/m^2^) median (IQR)	28.4 (1.7)	28.6 (1.3)	0.999
BMI-SDS median (IQR)	2.29 (0.14)	2.22 (0.17)	0.271
WC (cm) median (IQR)	90 (4.5)	92 (3)	0.646
WC/H median (IQR)	0.63 (0.02)	0.63 (0.01)	0.278
FM (%) median (IQR)	37.1 (2.1)	37.8 (2.1)	0.985
FM (Kg) median (IQR)	23.2 (3.6)	23.2 (2.7)	0.670
FFM (%) median (IQR)	62.8 (2.1)	62.1 (2.1)	0.913
FFM (Kg) median (IQR)	37.1 (3.4)	38.4 (3.2)	0.646
Weekly PA median (IQR) (CPAQ score) median (IQR)	1.6 (0.2)	1.6 (0.4)	0.569
Parent’s weekly PA (min/week) median (IQR)	1593 (413)	1656 (575)	0.484

BMI: body mass index; WC: waist circumference; H: height; FM: fat mass; FFM: fat free mass; PA: physical activity.

**Table 2:** Median values and interquartile ranges for anthropometric and body composition variables measured at baseline (T_0_) and at first (T_1_) and second appointment (T_2_) in the returners’ group with corresponding *p* values from Wilcoxon signed rank test.

Variable	T_0_	T_1_ (6 ± 3 Months after T_0_)	*p* (T_0_–T_1_)	T_2_ (12 ± 3 Months after T_0_)	*p* (T_1_–T_2_)	*p* (T_0_–T_2_)
	*n* = 451	*n* = 141		*n* = 79		
BMI (kg/m^2^)	28.6 (1.3)	27.4 (1.3)	<0.001	27.3 (1.4)	<0.001	0.002
BMI-SDS	2.2 (0.2)	2.0 (0.2)	<0.001	1.9 (0.3)	0.159	<0.001
WC (cm)	92 (3)	88.5 (4.5)	<0.001	88 (5)	0.092	<0.001
WC/H	0.63 (0.01)	0.60 (0.02)	<0.001	0.59 (0.03)	0.263	<0.001
FM (%)	37.8 (2.1)	35.2 (3)	<0.001	36.4 (4,9)	0.336	0.903
FM (kg)	23.2 (2.7)	22.4 (4.1)	<0.001	22.4 (6.6)	0.123	0.651
FFM (%)	62.1 (2.1)	63.8 (3)	<0.001	63.6 (4.3)	0.464	0.891
FFM (kg)	38.4 (3.2)	40.3 (5.3)	0.007	39.5 (8.9)	0.232	0.549

BMI: body mass index; SDS: standard deviation score; WC: waist circumference; H: height; FM: fat mass; FFM: fat free mass.

## Data Availability

The data presented in this study are available on request from the corresponding author.
